# Distal radius chondrosarcoma in a 35‐year‐old female patient: A case report

**DOI:** 10.1002/ccr3.8069

**Published:** 2023-10-23

**Authors:** Badaruddin Sahito, Hussain Haider Shah, Syeda Alishah Zehra, Javeria Saquib, Farea Ahmed, Muhammad Sheheryar Hussain, Tirth Dave

**Affiliations:** ^1^ Head of Orthopedics department Dr Ruth K M Pfau Civil Hospital Karachi Karachi Pakistan; ^2^ Dow University of Health Sciences Karachi Pakistan; ^3^ Dow Medical College Dr Ruth K M Pfau Civil Hospital Karachi Karachi Pakistan; ^4^ Bukovinian State Medical University Chernivtsi Ukraine

**Keywords:** chondrosarcomas, dedifferentiated chondrosarcoma, distal radius, elbow

## Abstract

**Key Clinical Message:**

Chondrosarcoma, although rare in the distal radius, poses significant challenges. Early diagnosis through incisional biopsy is essential. Surgical resection with margin control and fibular grafting can be effective, but vigilant surveillance is crucial due to its aggressive nature. Metastasis demands consideration of additional interventions or palliative care.

**Abstract:**

Chondrosarcomas constitute a rarity in the upper limbs, and their occurrence in the distal radius is even rarer with only one case previously documented. We report a case of distal radius chondrosarcoma in a 35‐year‐old female patient who presented with pain and swelling in her left wrist. Following an initial examination, an incisional biopsy was performed, confirming the diagnosis of dedifferentiated chondrosarcoma. The patient underwent a marginal resection of the distal radius and first carpal with ipsilateral fibular and locking compression plate fixation. Unfortunately, despite the interventions, the patient experienced recurrent swelling and ultimately required below‐elbow amputation, followed by above elbow amputation due to metastasis. Unfortunately, the patient passed away due to recurrence and metastasis.

## Graphical abstract

Radiological progression of dedifferentiated chondrosarcoma in the distal radius.

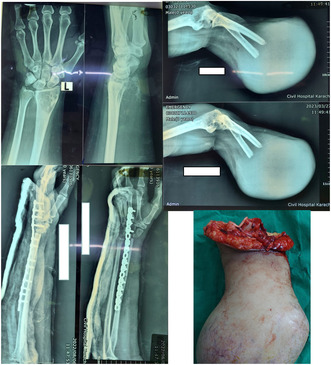

## 1 INTRODUCTION

Chondrosarcoma, the second most prevalent primary bone malignancy, is characterized by the formation of chondrocytes.^1^ Chondrosarcomas typically target adults, and the likelihood of occurrence increases as the patient's age increases.^2^ The pelvis is the most common origin location, followed by the proximal femur, proximal humerus, distal femur, and ribs.^3^

In terms of metastasis, acral lesions have a low likelihood of spreading, regardless of grade, while axial or proximal lesions are more prone to metastasize compared to tumors located in the distal extremities with similar histology.^1^ The occurrence of chondrosarcoma in the distal radius is exceptionally uncommon, with only one documented case reported to date.^4^ Chondrosarcoma can be classified into different types, ranging from slow‐growing, low‐grade tumors to aggressive, high‐grade forms.^5^ These malignant cartilage tumors include classical chondrosarcoma (grades I–III), dedifferentiated chondrosarcoma (grade IV), mesenchymal chondrosarcoma, and clear cell chondrosarcoma.^5^

Surgical resection is the primary and preferred treatment for individuals with localized chondrosarcoma because chondrosarcomas are resistant to both chemotherapy and radiotherapy due to the extracellular matrix, low percentage of dividing cells, and poor vascularity.^6^ However, reconstructive surgery options for the elbow can be complex due to the complexity of its anatomy and may result in disability.^4^

## 2 CASE PRESENTATION

**FIGURE 1:**
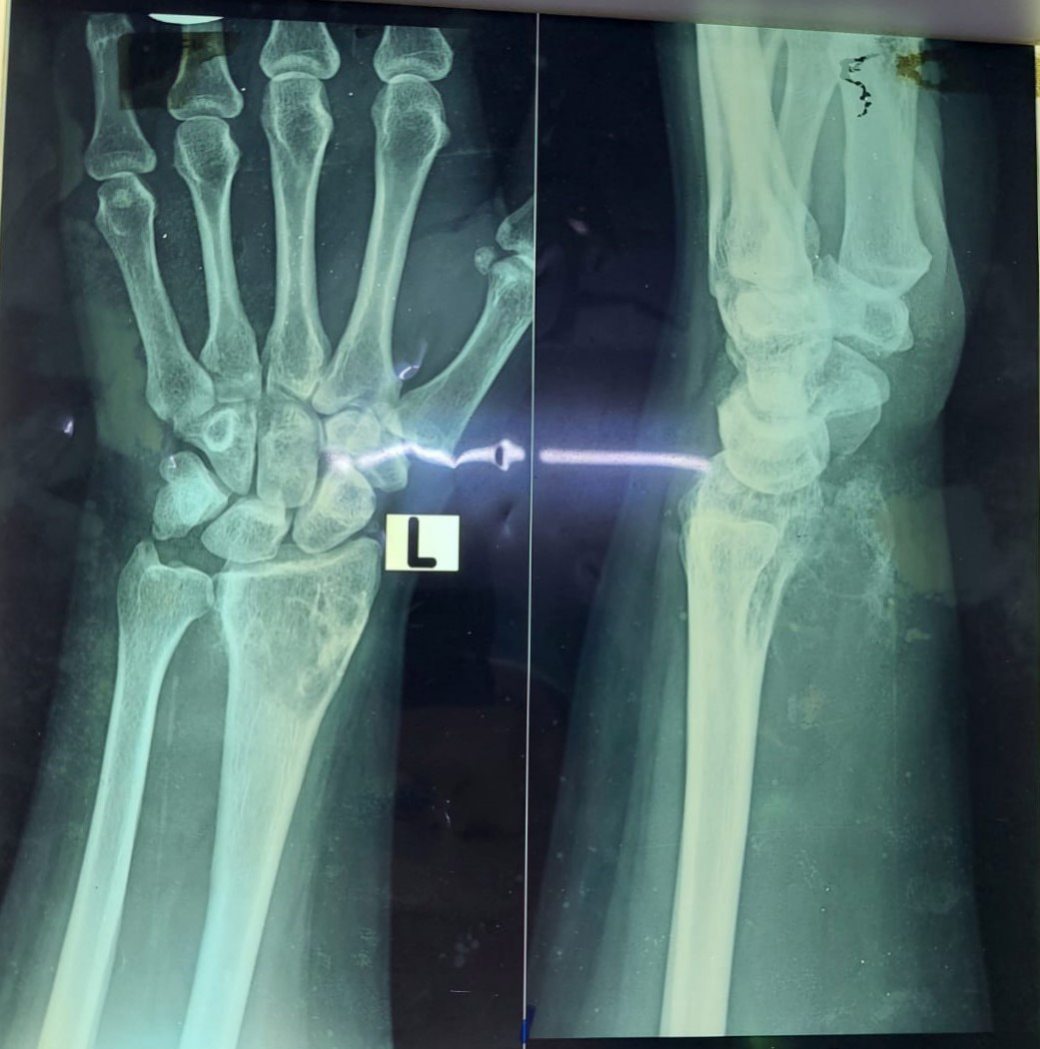
Osteolytic lesion involving distal radius with soft tissue extension volarly with cartilaginous matrix.

A 35‐year‐old female patient, with no known comorbidities or allergies, presented to Civil Hospital Karachi with a complaint of pain and swelling in her left wrist that had persisted for 9 months. The pain was characterized as mild to moderate in intensity, dull in nature, and worsened with hand movement. Concurrently, there was noticeable swelling in the left wrist, which rapidly increased in size over a period of 3 months. There were no other instances of swelling in the body, and the condition was not associated with weight loss. The patient has an unremarkable medical history and is married with two children. Upon examination, a 13 × 10 cm swelling extending from the forearm to the hand was observed in the left wrist. Dilated veins were visible on the anterior aspect of the wrist, while distal neurovascular function remained intact. X‐ray of the wrist AP and lateral view showed a mixed‐density lesion involving the distal radius, which extended densely into the soft tissue. The osteolytic lesion extended to the proximal carpal row (Figure 1).

MRI with contrast showed a hyperintense lesion on the T2 image and a hypointense on the T1. Bone scans show focal area of increased tracer uptake over the lower end of the right radius which corelates with the findings of the radiographs. The rest of the skeleton showed bilaterally symmetrical uptake in axial and appendicular skeleton. It revealed focal bone pathology involving the distal end of right radius, differential of Giant cell tumor was made, and further bone biopsy was advised.

### 2.1 Histology

Incisional biopsy concluded chondrosarcoma of distal radius which revealed fragments of hyaline cartilage, focally present bone, and skeletal muscle fibers. A few fragments of cartilage showed mild increased cellularity with occasional mitosis and binucleation. Features were suggestive of atypical cartilaginous tumor (dedifferentiated chondrosarcoma), while second biopsy report after the below‐elbow amputation showed skin covered tissue exhibiting a spindle cell neoplastic lesion arranged in fascicles and herring bone pattern. The cells were monomorphic spindle shaped with moderate atypia. Scattered dilated blood vessels were seen. Approximately 10/31 high‐power field (hpf) mitoses were identified with foci of necrosis. Areas of chondroid differentiation were not seen. Skin was tumor free. Immunohistochemical stains showed Negative ASMA, Desmin, S‐100, CD34, EMTLE‐1, STAT6, and focal positive CD99. The second biopsy in comparison with the first represented dedifferentiated chondrosarcoma.

### 2.2 Surgery

The plan was to do a marginal resection of the distal radius and fast carpal with ipsilateral fibular and LCP plate fixation. The patient was counseled about the procedure and potential complications, including nerve and vascular injury and recurrence. Under general anesthesia, the skin and subcutaneous tissue were incised through a dorsal approach. The tendon involved in the tumor was cut, the radial artery was ligated, and the median nerve was spared. The tumor with the distal radius in the proximal carpal row was marginally excised. The margins were negative.

**FIGURE 2:**
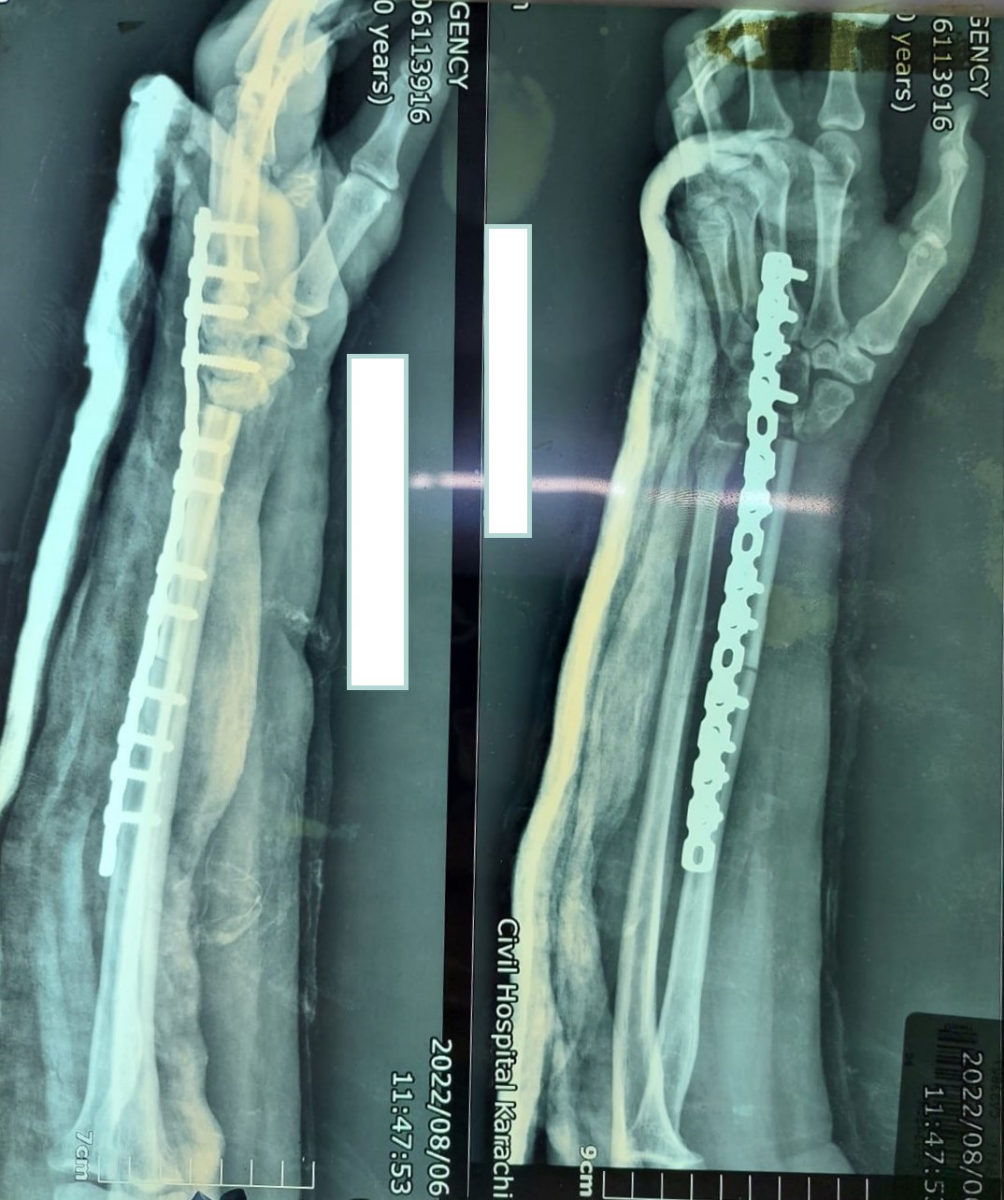
After marginal resection and reconstruction with fibular graft and locking recon plate.

Midshaft ipsilateral non‐vascularized fibular graft was taken through the lateral approach. The graft was placed at the distal radius, and the plate was fixed with a 20–30‐degree perpendicular bend using a recon plate (Figure 2).

**FIGURE 3:**
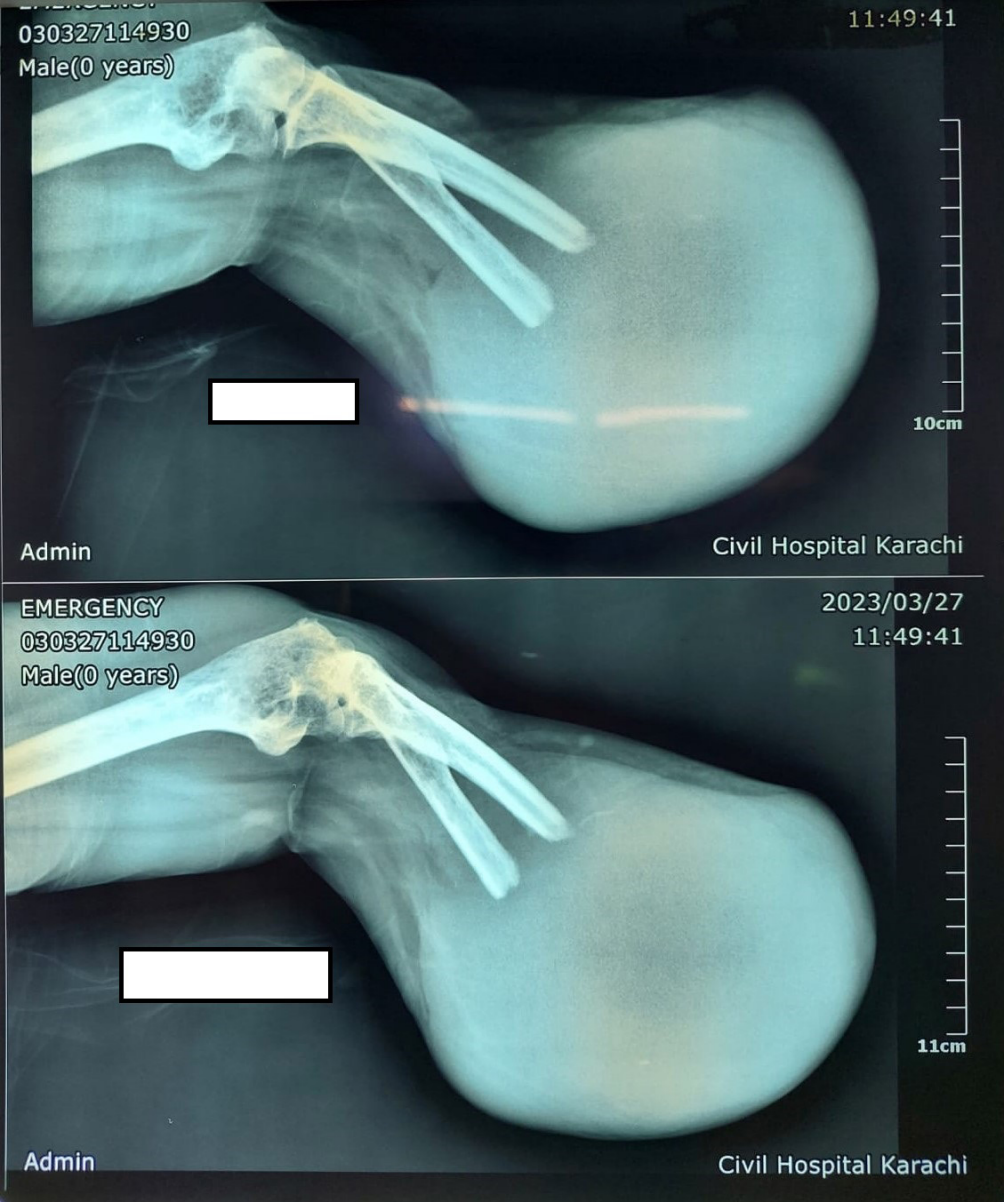
Recurrence after below‐elbow amputation.

**FIGURE 4:**
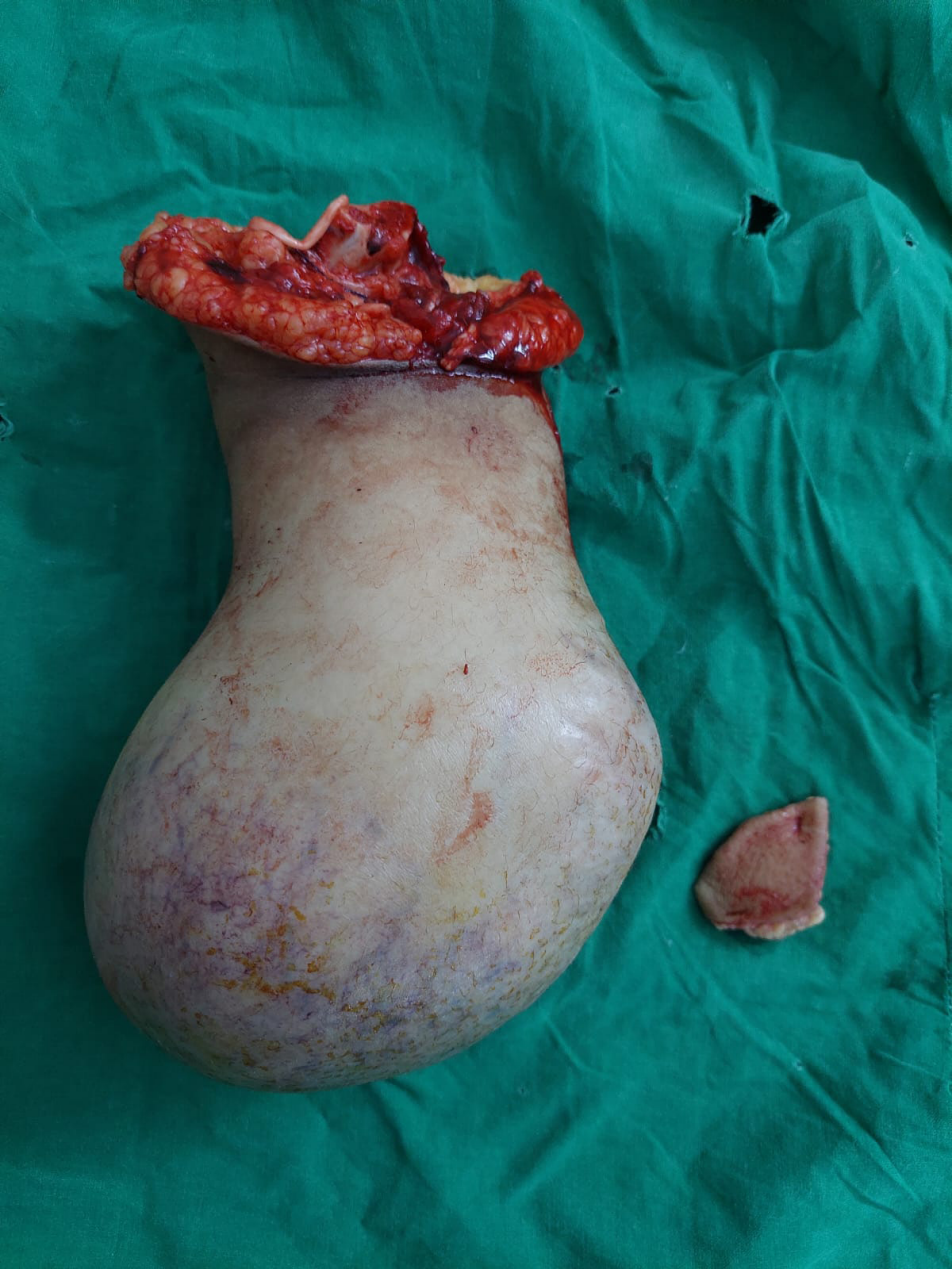
Recurrence after below‐elbow amputation was removed.

Following the surgery, the patient had an uneventful postoperative recovery. However, after a period of 5 months, the patient returned with a recurrence of swelling in her left distal radius. As a result, a below‐elbow amputation was performed. Unfortunately, 2 months following the below‐elbow amputation, the patient experienced a recurrence of swelling at the distal stump. As a result, an above elbow amputation became necessary to address the recurrent swelling. Further investigations were initiated, and notably, the patient also developed left‐side chest effusions and metastasis, indicating the dissemination of the disease to other parts of the body. She lost to follow‐up and did not went to the oncologist when she found has lung metastasis. No chemotherapy was initiated. Sadly, the patient passed away a few weeks later (Figures 3 and 4).

## 3 DISCUSSION

Chondrosarcomas are malignant mesenchymal tumors characterized by cartilage formation by tumor cells. They can be classified as primary chondrosarcomas, which arise independently, or secondary chondrosarcomas, which develop from preexisting enchondromas or osteochondromas.^6^ Unlike osteosarcoma, chondrosarcoma is less common during the first two decades of life. The mean age of patients diagnosed with chondrosarcoma typically falls between 35 and 45 years, and the male‐to‐female ratio is approximately 1.2:1.^6^ The age of our patient is 35, so it falls under the specified range.

Dedifferentiated chondrosarcoma (DDCS) was introduced by Dahlin and Beabout in 1971 and is applied to a high‐grade sarcoma present next to a low‐grade malignant cartilage‐forming tumor.^7^ DDCS accounts for approximately 11% of all chondrosarcoma cases and is associated with a poor prognosis.^8^ Grimer et al.^9^ reported that a majority of DDCS cases were found in the femur, humerus, and pelvis followed by axial skeleton. Chondrosarcoma of the hand and foot is a rare occurrence, and DDCS is an exceptionally rare tumor that affects the distal extremities.

Dedifferentiated chondrosarcoma has an aggressive nature and results in higher mortality rates compared to conventional chondrosarcoma or non‐cartilaginous sarcomatous components alone. The 5‐year survival rates for these are <20%.^9^, ^10^

Diagnosing chondrosarcoma can be challenging due to its potential resemblance to other benign or malignant bone lesions. In our case, the diagnosis was confirmed through an incisional biopsy, considered the gold standard for diagnosing bone tumors.^11^ The choice of treatment for chondrosarcoma depends on various factors, including the grade, size, location, and tumor stage.

Surgery remains the primary treatment modality, ranging from curettage and bone grafting to wide excision or amputation, depending on the specific circumstances. Radiation therapy and chemotherapy are less commonly employed and are typically reserved for high‐grade or recurrent tumors.^12^ A marginal resection is a surgical approach to remove a tumor with a minimal margin of healthy tissue, aiming to preserve functionality and minimize potential complications. This technique is typically employed for low‐grade or less aggressive tumors and may involve additional procedures such as bone grafting and internal fixation to restore stability and function.^13^ Another option is fibular grafting, which involves harvesting the fibula bone from the same leg and using it as a donor site for reconstructing the affected bone. This method is commonly used in orthopedic surgery for limb salvage purposes and has demonstrated favorable outcomes in bone union and restoration of functionality.^13^ Plate fixation, a form of internal fixation in which a metal plate and screws are utilized to stabilize bone fragments and facilitate healing, is often employed with bone grafting or fusion procedures. By providing immediate stability and support to the affected bone, plate fixation promotes proper alignment and enhances the potential for successful bone healing.

Unfortunately, despite the diligent efforts of the surgical team, this patient experienced a tumor recurrence shortly after the initial surgery, necessitating the amputation of the affected limb. This emphasizes the aggressive nature of chondrosarcoma and underscores the importance of vigilant surveillance and follow‐up care. The presence of metastasis in the chest indicates a poor prognosis for the patient, warranting consideration for additional treatment options or palliative care. During this challenging period, it is crucial to provide the patient and her family with thorough emotional and psychological support.

Our case underscores the importance of early detection and prompt medical intervention when dealing with rare and aggressive conditions like dedifferentiated chondrosarcoma. It is essential for healthcare providers to consider the possibility of such conditions in patients presenting with unusual or persistent symptoms. Advancements in imaging modalities, such as MRI, CT scans, and PET scans, may aid in early diagnosis. Research into novel therapies, including targeted therapies and immunotherapies, may offer hope for better outcomes in the future.

## 4 CONCLUSION

We report a case of recurrent chondrosarcoma of the distal radius in a 35‐year‐old female patient. The patient presented with diffuse swelling and pain in the left wrist, progressing rapidly. A bone scan revealed focal areas of heightened tracer uptake in the lower region of the left radius. Chondrosarcoma was suspected, with a giant cell tumor considered a differential diagnosis. The diagnosis of chondrosarcoma was ultimately confirmed through histopathology.

## AUTHOR CONTRIBUTIONS

**Badaruddin Sahito:** Conceptualization; writing – original draft; writing – review and editing. **Hussain Haider Shah:** Data curation; writing – original draft; writing – review and editing. **Syeda Alishah Zehra:** Writing – original draft; writing – review and editing. **Javeria Saquib:** Writing – original draft; writing – review and editing. **Farea Ahmed:** Writing – original draft; writing – review and editing. **Muhammad Sheheryar Hussain:** Writing – original draft; writing – review and editing. **Tirth Dave:** Supervision; writing – original draft; writing – review and editing.

## FUNDING INFORMATION

This research received no specific grant from any funding agency in the public, commercial, or not‐for‐profit sectors.

## CONFLICT OF INTEREST STATEMENT

None declared.

## ETHICS STATEMENT

The ethical approval was not required for the case report as per the country's guidelines.

## CONSENT

Written informed consent was obtained from the patient to publish this report in accordance with the journal's patient consent policy.

## Data Availability

The data that support the findings of this article are available from the corresponding author upon reasonable request.
